# The Synthesis and Analysis of the Cytotoxicity of Al_2_O_3_-Supported Silver Nanoparticles Prepared by the Plasma Chemical Process Initiated by Pulsed MW Radiation in the Al_2_O_3_–Ag Powder Mixtures

**DOI:** 10.3390/ijms25105326

**Published:** 2024-05-14

**Authors:** Nina N. Skvortsova, Nailya S. Akhmadullina, Ildar Yu. Vafin, Ekaterina A. Obraztsova, Yanislav S. Hrytseniuk, Arina A. Nikandrova, Dmitrii A. Lukianov, Tatiana E. Gayanova, Elena V. Voronova, Oleg N. Shishilov, Vladimir D. Stepakhin

**Affiliations:** 1 A.M. Prokhorov General Physics Institute of the Russian Academy of Sciences, Vavilova st. 38, Moscow 119991, Russia; mukudori@mail.ru (N.N.S.); ildar@fpl.gpi.ru (I.Y.V.); brenka@mail.ru (E.A.O.); tatyanagayanova97@gmail.com (T.E.G.); oshishilov@gmail.com (O.N.S.); scooter11@mail.ru (V.D.S.); 2 A.A. Baikov Institute of Metallurgy and Material Science of Russian Academy of Sciences, Leninsky av. 49, Moscow 119991, Russia; 3 Faculty of Chemistry, Moscow State University, Leninskie Gory, Moscow 119991, Russia; gritseniuk2000@yandex.ru (Y.S.H.); arinanikandrova@mail.ru (A.A.N.); or dlukianov@icloud.com (D.A.L.); 4 M.V. Lomonosov Institute of Fine Chemical Technologies, MIREA—Russian Technological University, Vernadskogo av. 86, Moscow 119571, Russia

**Keywords:** aluminum oxide, silver, plasma chemical process, microwave radiation, cytotoxicity

## Abstract

An original plasma chemical process initiated by microwave discharge in a mixture of metal and dielectric powders was applied to prepare specific materials, which consisted of microsized spherical particles of aluminum oxide covered with silver nanoparticles. The prepared materials are highly uniform in shape, size distribution, and composition. Their cytotoxicity was investigated using the human cell lines MCF7, HEK293T, A549, and VA-13 and the bacterial strains *E. coli* JW5503 (ΔtolC) and *E. coli* K12. Their cytotoxicity was found not to exceed the cytotoxicity of the starting materials. Thus, the prepared materials can be considered highly promising for catalysis and biotechnology applications.

## 1. Introduction

In recent decades, nanomaterials, particularly metal nanoparticles, have found wide applications in various industries. For any widely used material, safety is always a focus and potential toxicity toward living cells is one of the key factors for that. The cytotoxicity of nanomaterials is a current topic for many researchers, especially those dealing with materials for medical purposes [1,2,3]. Among the metals used by the industry in nanomaterials, silver is known to have a high toxicity toward many microorganisms [4,5] and viruses [2,6,7]. The wide application areas of silver in the industry [8,9,10,11,12,13,14] require deep knowledge of its potential toxicity in every specific case. In general, the toxicity depends on the metal’s form, oxidation state, solubility, and concentration [2,4]. For silver nanoparticles, it has been shown that their toxicity is hardly affected by their shape, size, and crystal structure [3,4]. In some cases, cytotoxicity has been studied as a function of nanoparticles’ concentration and exposure time. For example, Ranjan and co-workers found [15] that the level of lipid peroxidation significantly increased after 24 h of treatment even at a silver concentration of 10 μg/mL (3.61 ± 0.15 nmol hydroperoxide/mg protein). The particle size was below 60 nm in that study. Usually, cytotoxicity increases with a decreasing particle size [16,17,18,19,20,21]. Also, the influences of the shape, surface features, and crystallinity of silver nanoparticles on their cytotoxicity have been particularly mentioned in many studies [22,23,24].

Nowadays, there are several approaches for the preparation of metal nanoparticles supported by ceramic materials, e.g., metal oxides and nitrides, zeolites, etc. They include precipitation and co-precipitation methods, the cold plasma method, the laser electrodeposition method, the immobilization of ultrafine metal nanoparticles on high-surface-area materials, etc. [25,26,27,28,29,30]. In the last decade, we have developed a new plasma chemical method for the preparation of metal nanoparticles supported on the surfaces of microparticles of oxides and oxynitrides [31,32]. To date, we have already succeeded in the preparation of platinum and palladium nanoparticles immobilized on the surfaces of aluminum, silicone, and titanium oxide for potential use as heterogeneous catalysts [33]. The developed approach is based on the ability of a microwave (MW) pulse generated by a high-power gyrotron to initiate autothermic plasma chemical chain reactions in the mixtures of metal and dielectric powders, which also involve the gas phase [34]. Chain reactions are initiated when the breakdown energy threshold is exceeded. The threshold depends on the nature of the powder and the metal powder content [35]. When the chain reaction process is initiated, the microwave breakdown at the contact points between the metal and dielectric particles leads to the scattering of the particles, molecules, atoms, and ions into the volume of the reactor. Then, the formation of new materials occurs in the plasma and gas medium at atmospheric pressure. Microparticles of new chemical compositions (including oxides, nitrides, and oxynitrides) are formed as a result of highly non-equilibrium plasma chemical processes, which are 2–3 orders of magnitude longer than the pulse itself. Metal nanoparticles are formed on the surfaces of the microparticles. The composition of the particles and their size distribution (from dozens nm to dozens microns) are determined by the process conditions and are well-reproducible [33,36,37].

Herein, we describe the synthesis, morphology, and cytotoxicity of the nano- and microparticles formed in the plasma chemical process initiated by microwave pulses in mixtures of silver metal and aluminum oxide powders for catalytic applications. Earlier, the same approach was applied to estimate the toxicity of nano- and microparticles synthesized from mixtures of titanium and boron or boron nitride powders (Ti + B, Ti + BN*_cub_*, and Ti + BN*_hex_*). The bioactivity was estimated utilizing a bacterial cell model in vitro [38].

## 2. Results and Discussion

### 2.1. Synthesis of Al_2_O_3_-Supported Silver Nanoparticles

**Figure 1:**
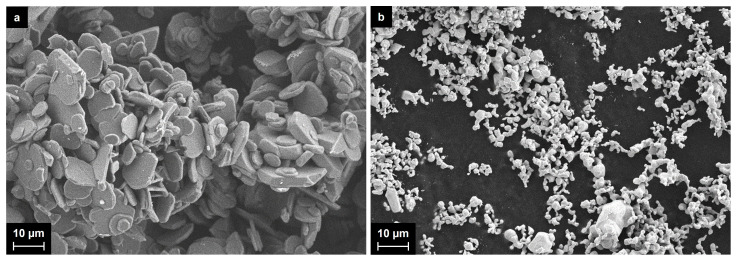
(**a**) SEM image of the starting aluminum oxide powder; (**b**) SEM image of the starting silver powder.

Al_2_O_3_-supported silver nanoparticles were synthesized by the plasma chemical process initiated by pulsed MW radiation in mixtures of aluminum oxide and silver powders. The silver powder prepared as described in Section 3.1 was mixed with aluminum oxide, and the silver content ranged from 2 wt.% to 20 wt.%. Both the powders of aluminum oxide and silver were studied with SEM, and the typical images are given in Figure 1.

The starting aluminum oxide powder consists of plane particles of irregular shapes, which were observed to be up to 50 microns in size. The initial silver particles were also mainly irregular in shape, although some of them were considered to be slightly distorted spheres. Their size ranged mainly from 1–2 to 10 microns, and there were also a few larger agglomerates.

The mixtures were prepared by the vigorous grinding of the components for 10 min in an agate mortar with the addition of some acetone, followed by air drying at 120 °C for 30 min. Then, the mixtures were treated with pulses of MW radiation with a duration of 2 to 6 ms and a power of 200 to 400 kW. In a typical experiment, 3.0 g of a mixture was initially treated with a 2 ms/200 kW pulse. If there was no breakdown, and the plasma chemical process was not initiated, then the MW power was increased by 50 kW up to 400 kW, and the pulse duration was increased by 2 ms up to 8 ms until breakdown was observed. If the longest pulse with the highest power was still not sufficient, then an initiator was set up in the reactor (see Section 3.2) to produce the so-called non-self-sustaining discharge [39], which then helped initiate the plasma chemical process. The initiator used was a stainless-steel sponge. Table 1 summarizes the conditions that led to the development of the plasma chemical process and the formation of Al_2_O_3_-supported Ag nanoparticles.

As expected, the mixtures with low silver contents (2 wt.% and 5 wt.% for entries 1 and 2) required the presence of the initiator to start the plasma chemical process via the non-self-sustaining discharge. Meanwhile, in the mixtures with silver contents of 10 wt.% and 20 wt.%, the energy threshold was ~0.8 kJ/g, and no initiator was needed.

**Figure 2:**
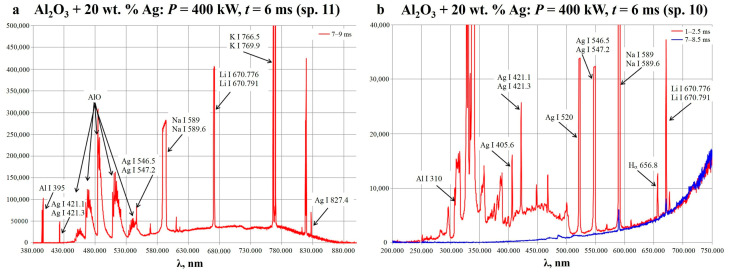
The typical optical emission spectra obtained from the free volume of the reactor ((**a**), spectrometer 11) and the bottom side of the powder ((**b**), spectrometer 10) for the Al_2_O_3_ + 20 wt.% Ag mixture.

The breakdown led to the development of the plasma chemical process, which has been described in detail earlier for Al_2_O_3_ + Al mixtures in [40]. All three main stages (initiation, the main stage, and decay) were observed, which allowed us to monitor the process with optical emission spectroscopy. The spectra were obtained from the free volume of the reactor and the bottom side of the powder mixture (spectrometers 11 and 10—see Section 3.2). The typical spectra obtained for the Al_2_O_3_ + 20 wt.% Ag mixture are given in Figure 2.

In the given spectra, the following bands were attributed: the bands of atomic aluminum (Al I 310 nm; 395 nm), the bands of the AlO molecule (450–550 nm), the single bands of atomic silver (Ag I 405.6 nm; 520 nm; and 827.4 nm), the unresolved double bands of atomic silver (Ag I 421.1 nm; 421.3 nm; and 546.5 nm, 547.2 nm), the H_α_ band at 565.8 nm, and the bands of alkali metals including the saturated double bands of atomic sodium (Na I 589 nm; 589.6 nm), lithium (Li I 670.776 nm; 670.791 nm), and potassium (K I 766.5 nm; 769.9 nm). Since alkali metal bands are usually highly intense due to the effective ionization of these metals, they can be considered, rather, as impurities. The sodium and hydrogen may have come from sodium borohydride, which was used for the preparation of the silver powder. Another probable source of hydrogen was atmospheric water. Sodium, lithium, and potassium are regular impurities in metal oxides, including aluminum oxide, and may have come from the glassware as well.

From the recorded amplitudes of sections of the continuous spectrum free from atomic lines and molecular bands, the Planck temperature of the bottom surface of the powder mixture was determined by Wien’s displacement law [41]. The temperature was calculated to be 1500–1350 ± 200 K during the first 8 ms of the plasma chemical process. Although the found temperature was far below the boiling points of both aluminum oxide and silver, one must keep in mind that the temperature of the plasma phase was obviously much higher. Even that temperature is enough to form AlO molecules and free silver atoms. As a result, the plasma chemical process conditions could have led to changes in the aluminum oxide surface composition and also to the transfer of silver from the silver microparticles to the surface of the aluminum oxide via the gas phase, which was confirmed by the analysis of the products.

**Figure 3:**
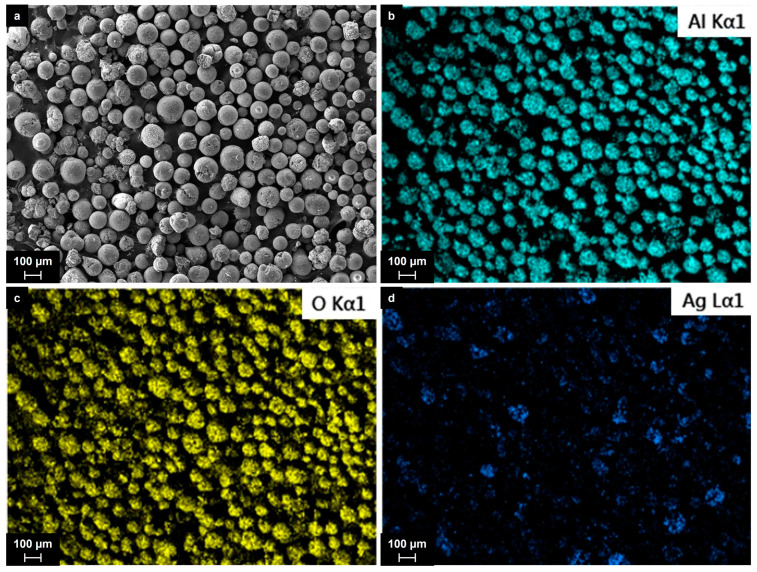
SEM image (**a**) and elemental maps (**b**–**d**) of the particles collected from the wall of the quartz cylinder after the treatment of the Al_2_O_3_ + 5 wt.% Ag mixture.

The products of the plasma chemical process were collected from the walls of the quartz cylinder set inside the reactor. Although almost all the mixture seemed to be involved in the process, the particles from the plasma and the gas phase, however, were supposed to be more representative. Their total amount varied from 50 to 200 mg, which was enough for all further studies. Figure 3 shows the typical SEM images and the results of the elemental mapping analysis of the collected material after the treatment of the Al_2_O_3_ + Ag mixture.

**Figure 4:**
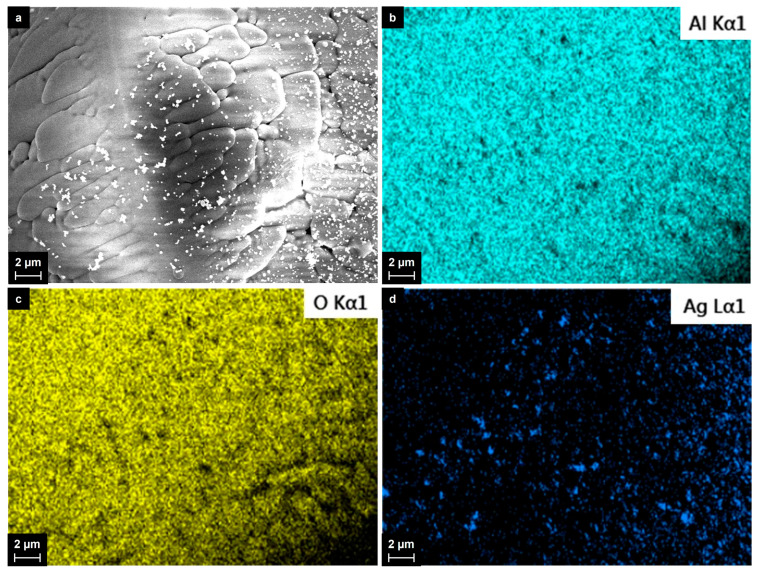
Detailed SEM image (**a**) and elemental maps (**b**–**d**) of the particles collected from the wall of the quartz cylinder after the treatment of the Al_2_O_3_ + 5 wt.% Ag mixture.

The material’s appearance was completely different from that of the initial powders, which contained plane flakes with sharp edges. For all silver contents, the material consisted of spherical particles, whose composition was related to the aluminum oxide according to the EDX spectroscopy data and the elemental mapping analysis. The Al_2_O_3_ particles varied in size from 20 to 150 microns, mainly from 70 to 120 microns. A detailed image of the Al_2_O_3_ particles with the results of the elemental mapping analysis is given in Figure 4. Most of the spherical Al_2_O_3_ particles were covered with nanosized silver particles, which appear as dark areas in the aluminum and oxygen maps. The individual silver particles ranged in size mainly from 50 to 200 nm and were spherical in shape. The larger silver particles (up 1–2 microns in size) had irregular shapes and were most likely agglomerates. We did not observe even larger particles consisting of silver in the collected materials.

### 2.2. Cytotoxicity of Al_2_O_3_-Supported Silver Nanoparticles against Human Cells and Bacteria

To assess the nanoparticles’ cytotoxicity, we selected cells representing both the eukaryotic and prokaryotic domains of living organisms. Among eukaryotes, human cells were selected as the most important, and among prokaryotic cells, the bacterium *E. coli* was selected as quite significant, widespread, and the most studied model microorganism.

Samples of particles from the starting and resulting mixtures were dispersed in water (20 g/L) and treated with ultrasound for 10 min (five series of 2 min). It should be noted that the particles of the Al_2_O_3_ + Ag mixtures sedimented faster than the particles of the Ti + B and Ti + BN mixtures tested earlier according to the same protocol [38]. This made it harder to estimate the local concentration since the cells were also affected by the sedimentation of the substance deposited directly on the used adhesion cells.

**Figure 5:**
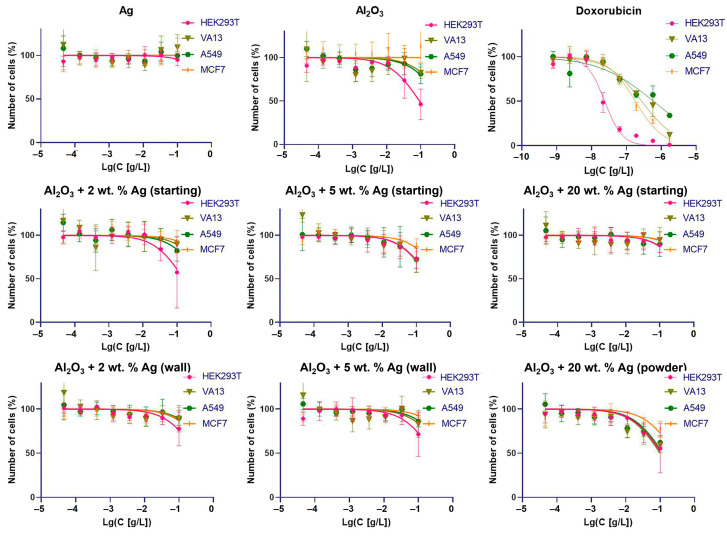
Concentration–viability dependencies for cells treated by studied substances. Doxorubicin is given for comparison. Results for the initial Al_2_O_3_ and Ag powders are shown in the top row. Middle row: starting mixtures containing 2 wt.%, 5 wt.%, and 20 wt.% of silver. Bottom row: the mixtures treated with MW radiation, and samples collected from the walls of the quartz tube (marked as ‘wall’) and from the surface of the powder in the reactor (marked as ‘powder’).

We assumed that the initial aluminum oxide and silver powders did not have significant cytotoxicity due to their widespread use in medicine. Figure 5 presents the results of the cytotoxicity analyses of the substances obtained from the Al_2_O_3_ + Ag mixtures and collected from different areas of the reactor. The testing of the cytotoxicity of each sample in human cells was carried out for 72 h, in a series of eight dilutions with concentrations of 0.05–100 mg/L. The half-maximum inhibition concentration (IC50_abs_) was not achieved for all four cell lines used: MCF7′ and A549 tumor cells, etiologically non-tumor human HEK293T cells, and immortalized VA-13 fibroblasts. Taking into account small effects at maximum concentrations, it can be concluded that at the level of the cellular models, the cytotoxicity of the obtained materials did not exceed the cytotoxicity of the starting substances.

**Figure 6:**
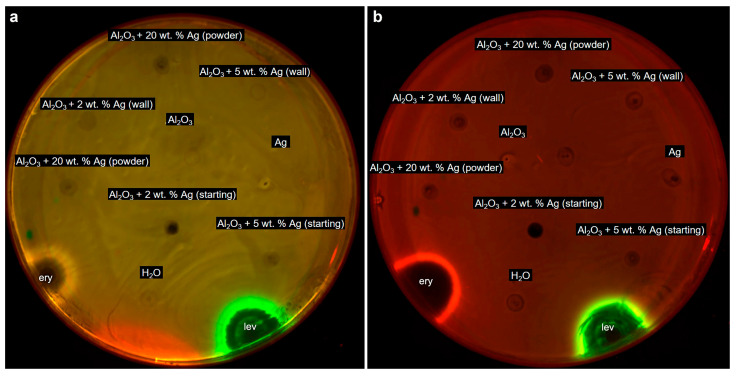
Evaluation of the bacterial toxicity of the particles toward (**a**) the hypersensitive strain of *E. coli* JW5503 (ΔtolC, with impaired efflux) and (**b**) the standard strain of *E. coli* K-12. The figure shows the starting mixtures containing 2 wt.%, 5 wt.%, and 20 wt.% of silver and the mixtures treated with MW radiation, the samples collected from the walls of the quartz tube (marked as ‘wall’) and from the surface of the powder in the reactor (marked as ‘powder’), and the control antibiotics («ery» is erythromycin, and «lev» is levofloxacin).

Then *E. coli* strains K-12 and JW5503 (ΔtolC, with an impaired efflux system) were used to evaluate the bacterial toxicity. Figure 6 illustrates the test results. As one can see, bacterial growth inhibition areas are visible near the places where the antibiotics samples were dropped, which is not observed for the particle samples. The antibiotics used as controls were erythromycin at 5 mg/mL (the red edge around the inhibition zone corresponds to the gene expression of the fluorescent protein Katushka2S induced by the disruption of protein biosynthesis in the bacterial cell) and levofloxacin at 25 ng/mL (the green edge around the inhibition zone corresponds to the gene expression of the fluorescent protein TurboRFP induced by DNA damage). Moreover, the activity of the particles was evaluated using a suspension culture of *E. coli* cells. For all the samples, the minimum inhibitory concentration was determined to be more than 200 µg/mL, no matter what the silver concentration was in the initial mixture. Therefore, the synthesized particles did not cause the inhibition of growth nor death of the *E. coli* strains JW5503 (ΔtolC) and K-12 in both bacterial cell tests.

## 3. Materials and Methods

### 3.1. Raw Materials

All the reagents were chemical-grade (≥99%) and used as supplied (by “Khimmed LLC”, Moscow region, Russia) unless otherwise specified. The particle size for the aluminum oxide powder mainly ranged from ~0.5 microns to ~15 microns. The silver powder was prepared by the reduction of a silver nitrate water solution (1 M) with sodium borohydride NaBH_4_ in an alkali medium (NaOH; pH ~11–12) at 50 °C for 1 h. The obtained solid was washed with deionized water and dried at 80 °C. Both the aluminum oxide and silver powders were studied with SEM, and the results are given below.

### 3.2. Plasma Chemical Synthesis

**Figure 7:**
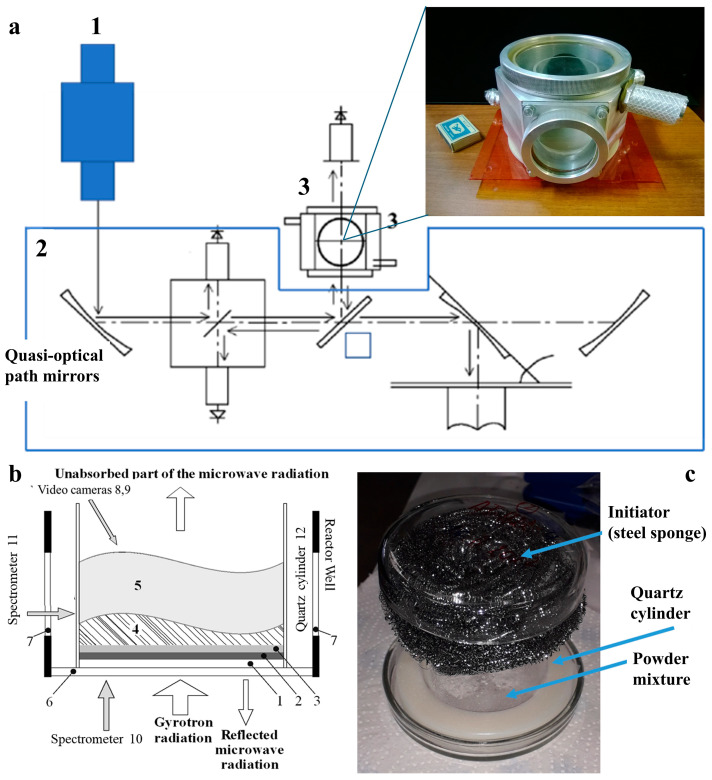
(**a**) General scheme of the plasma chemical setup for the treatment of the mixtures of powders with pulses of microwave radiation: 1—high-power gyrotron (0.8 MW/75 GHz), 2—quasi-optical pathway, and 3—plasma chemical reactor; (**b**) 1—quartz plate, 2—isolating layer of dielectric powder (optional), 3—reaction mixture, 4—gas phase, 5—plasma phase, 6 and 7—bottom- and side-view windows, 8—high-speed camera, 9—regular optical camera, 10 and 11—AvaSpec optical emission spectrometers (operating in the range of 370 ÷ 920 nm with a 0.7 nm resolution, and in the range 250 ÷ 800 nm with a 0.8 nm resolution, respectively, obtaining 100 spectra at 4 ms intervals), and 12—quartz cylinder; and (**c**) image of the initiator (stainless-steel sponge) on the quartz cylinder.

Plasma chemical synthesis was carried out utilizing a specially designed setup, which included a high-power gyrotron, a quasi-optical pathway, a plasma chemical reactor, and a diagnostic complex. A detailed description of the setup is given in refs. [34,35,36,37,38,40]. The gyrotron generated pulses of microwave radiation, which varied from 2 to 8 ms in duration and from 150 to 400 kW in power. The pulses were directed by the quasi-optical pathway into the plasma chemical reactor, where the breakdown happened, and the plasma chemical processes occurred. The processes occurred in the free volume of the reactor and resulted in the formation of new nano- and microparticles, which fell back onto the bottom of the reactor and deposited on the walls of the quartz cylinder (Figure 7). The diagnostic complex allowed us to measure the initial power of the pulse as well as the power of the beam passing through the reactor and to collect the optical spectra from different parts of the reactor.

### 3.3. Composition and Morphological Analyses

Measurements were carried out using a Zeiss Merlin scanning electron microscope (SEM). Accelerating voltages from 1 to 2 kV were used, which made it possible to obtain high-quality images of the obtained particles in the magnification range from 100 to 30,000 times. The samples were fixed on the surface of a conductive carbon adhesive film and placed on the stage of the electron microscope. The conductive carbon film provided the reliable fixation of the test substance particles while forming a uniform background that did not interfere with the studied structures.

The microscope was equipped with an Oxford Instruments INCA x-act X-ray microanalysis attachment. Energy-dispersive X-ray spectroscopy and mapping were carried out on the studied samples to access the chemical compositions and homogeneity of the obtained samples. To register the EDX data, increased accelerating voltages of 10–15 kV were used.

### 3.4. Cytotoxicity Assay Protocols

The samples were tested for cytotoxicity without separation into fractions, as mixtures of micro- and nanosized particles. The suspension of the particles for the biological tests was carried out with an ultrasonic probe-based 130 W 20 kHz CPX130PB system with an 80% amplitude (Cole-Palmer Instruments, Vernon Hills, IL, USA) for 15 s of processing with a gap of a few minutes for cooling.

#### 3.4.1. Cytotoxicity Tests against Human Cells (MTT Test)

The cytotoxicity of the studied substances was tested using the MTT (3-(4,5-dimethylthiazol-2-yl)-2,5-diphenyl tetrazolium bromide) assay [42] with some modifications. A total of 2500 cells per well for the MCF7 and HEK293T cell lines, 3000 cells per well for the A549 cell line, and 4000 cells per well for the VA-13 cell line were plated in 135 µL of the DMEM-F12 medium (Gibco, ThermoFisher Scientific, Norristown, PA, USA) in a 96-well plate and incubated in a 5% CO_2_ incubator for the first 16 h without treatment. Then, 15 µL of each suspension of the tested substances in the medium was added to the cells. The cells were treated for 72 h with eight dilutions of our substances (each performed in triplicate) and doxorubicin as a control substance. The MTT reagent (Paneco LLC, Moscow, Russia) was then added to the cells up to a final concentration of 0.5 g/L (a 10X stock solution in PBS was used), and the cells were incubated for 2 h at 37 °C in the incubator under an atmosphere of 5% CO_2_. The MTT solution was then discarded, and 140 µL of DMSO (PharmaMed LLC, Russia) was added. The plates were swayed in a shaker (80 rpm) to dissolve the formazan. The absorbance was measured using a microplate reader (VICTOR X5 Light Plate Reader, PerkinElmer, Shelton, CT, USA) at a wavelength of 565 nm (in order to measure the formazan concentration). The results were used to construct dose–response graphs and to calculate the IC50_abs_ values (the IC50_abs_ is the concentration resulting in a two-fold decrease in the number of cells in comparison with untreated cells) with GraphPadSoftware, Inc., San Diego, CA, USA.

#### 3.4.2. Cytotoxicity Tests against *E. coli* Cells

The *E. coli* strains JW5503 (ΔtolC) [43] and K-12 (wt) transformed with the pdualrep2 reporter plasmid [44] were used to evaluate the mechanism of antimicrobial action and activity against bacteria. The overnight cultures of the reporter strains were diluted with fresh LB medium to an optical density of 600 nm of 0.05–0.1. Then, the cultures were plated on LB-agar plates coated with 100 mg/mL of ampicillin. On the agar plates coated with the reporter strains, 2 µL of each compound at a concentration of 20 mg/mL was spotted. Erythromycin (Ery; 5 mg/mL) and levofloxacin (Lev; 25 mg/mL) were used as control antibiotics on an agar plate. After 12 h of incubation at 37 °C, the agar plates were scanned with ChemiDoc (Bio-Rad, Moscow, Russia) in the Cy3 (TurboRFP) and Cy5 (Katushka2S) channels to determine the zones of inhibition and fluorescence levels of the reporter proteins.

The minimum inhibitory concentration (MIC) in the LB medium was determined by microdilution analysis, as described in a previous publication [45]. In short, the cell concentration was adjusted to ~5 × 10^5^ cells/mL. The test compounds were serially diluted two times, starting at 400 µg/mL, in a 96-well microplate (100 µL/well). The microplates were incubated at 37 °C under shaking. The optical density was measured at λ = 600 nm in each well, and the lowest concentration of each test compound that did not result in bacterial growth after 16–20 h was reported as the MIC.

## 4. Conclusions

In the last decade, we have developed a new plasma chemical method for the preparation of metal nanoparticles supported on the surfaces of microparticles of oxide and oxynitrides. The developed approach is based on the ability of microwave pulses generated by a high-power gyrotron to initiate autothermic plasma chemical chain reactions in mixtures of metal and dielectric powders, which also involve the gas phase. Herein, we successfully applied the developed approach for the preparation of Al_2_O_3_-supported silver nanoparticles. The treatment of the Al_2_O_3_ + Ag powder mixtures with silver contents ranging from 2 wt.% to 20 wt.% led to the formation of spherical aluminum oxide particles with a size of ~100 microns covered with silver particles, which were found to be from 50 nm to 1–2 microns in size. The prepared micro/nanosized structures showed high uniformity in their shapes, size distributions, and compositions, which makes them promising materials for catalysis and biotechnology applications.

The cytotoxicity of the new materials was assessed by testing them in the tumor cell line MCF7 and etiologically non-tumor human HEK293T cells; additionally, tests were carried out in the tumor epithelial cell line of the lungs A549 and the immortalized VA-13 fibroblasts. Furthermore, it was proved that the particles did not cause harm to the two *E. coli* strains, the hypersensitive ΔtolC and the wild-type K12. Well-known antibiotics were used as controls.

It was found that none of the tested samples caused cytotoxicity. This gives us a basis to claim that the cytotoxicity of the synthesized materials was not higher than that of the parent substances in the cell line models. Silver particles are known to have antibacterial properties [4]. Considering that the new compounds were not cytotoxic to the *E. coli* JW5503 (ΔtolC) and *E. coli* K12 strains, it can be concluded that the particles are not harmful to the human microbiome. In appropriate conditions, silver particles may have wider applications than have been accepted so far. It would be interesting to conduct further studies in this area.

To summarize, the prepared materials were confirmed to be safe and are considered to have potential for a broad range of applications.

## Figures and Tables

**Table 1:** Conditions for the development of the plasma chemical process in the Al_2_O_3_ + Ag mixtures.

#	Silver Content, wt.%	Pulse Duration, ms	Pulse Power, kW	Specific Energy *, kJ/g	Initiator
1	2.0	6	300	0.6	Yes
2	5.0	6	400	0.8	Yes
3	10.0	6	400	0.8	No
4	20.0	6	400	0.8	No

* Specific energy = (pulse duration × pulse power)/sample weight. The sample weight was 3.0 g in a typical experiment, e.g., for entry 1, specific energy = (6 × 10^−3^ s × 3 × 10^5^ W)/3 g = 6 × 10^2^ J/g (0.6 kJ/g).

## Data Availability

The data are contained within this article.
